# Rebound iritis with a well-circumscribed anterior chamber fibrin mass after uncomplicated cataract surgery

**DOI:** 10.1186/s12348-021-00270-2

**Published:** 2021-10-12

**Authors:** Nilesh Raval, Wen-Jeng (Melissa) Yao, Gene Kim, Joann J. Kang

**Affiliations:** Department of Ophthalmology, Albert Einstein College of Medicine, Montefiore Medical Center, 3332 Rochambeau Ave, 3rd Floor, Bronx, NY 10467 USA

## To the editor

We report a case of subacute rebound iritis characterized by a globular, pedunculated anterior chamber mass that resolved after topical steroid burst.

## Case report

A 59-year-old female with no significant past ocular history underwent cataract extraction (CE) with phacoemulsification and posterior chamber intraocular lens (PCIOL) insertion in the right eye (OD). Intraoperatively, a Malyugin ring was deployed due to poor dilation, however the remainder of the surgery was uneventful. On the first postoperative day, 3+ mixed cell and pigment in the anterior chamber (AC) without fibrin reaction was observed, which resolved by the second week with topical steroid administration. The patient was lost to follow up and was non-compliant with her steroid taper.

**Fig. 1:**
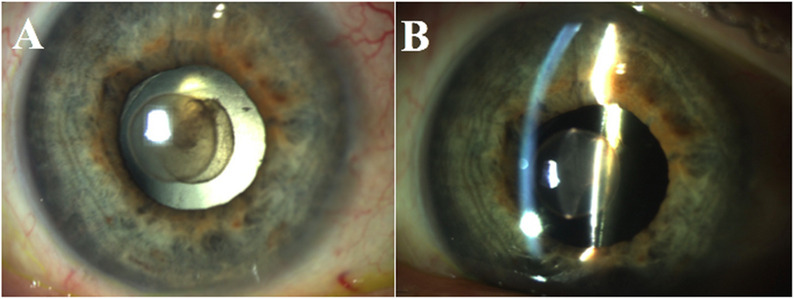
Sharply-circumscribed, globular, partially opaque mass with a round, smooth border and a well-demarcated stalk attached to the anterior surface of the PCIOL in retro-illumination (**A**) and direct illumination (**B**)

Eight weeks later, the patient presented with eye pain OD. Visual acuity (VA) was 20/40 and intraocular pressure (IOP) by Goldmann applanation was 6; there was no relative afferent pupillary defect (rAPD) noted. Slit lamp biomicroscopy was remarkable for trace Descemet’s folds (DF), 3+ mixed AC cell and pigment, and a sharply-circumscribed, globular, partially opaque anterior chamber mass with smooth borders and a well-demarcated stalk attached to the surface of the PCIOL (Fig. 1A and B). The PCIOL was well-centered and dilated fundus examination was unremarkable.

**Fig. 2:**
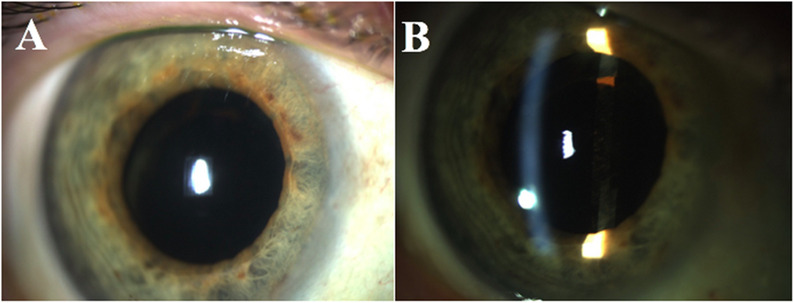
Complete resolution of the AC mass after three days of topical steroids (**A**, **B**)

The patient was started on prednisolone acetate 1% eye drops every two hours and cyclopentolate 1% eye drops twice a day. Examination three days later showed complete resolution of the mass (Fig. 2A and B) and improvement in AC inflammation to 1+ cell. The patient endorsed improvement in her symptoms and was discharged on a tapering regimen of topical steroids but was again lost to follow-up.

## Discussion

The post-operative anterior segment mass reported above most likely represents a fibrinous exudate secondary to rebound iritis. Rebound and persistent iritis are well-known entities that may occur after cataract surgery. Neatrour et al [1] reported that pupil expansion devices significantly increase the risk of persistent (> 1 month) post-operative iritis.

Fibrinous exudates are occasionally encountered after intraocular surgery, more commonly after pars plana vitrectomy [2–5]. Fibrin reaction has also been reported after anterior segment surgery involving iris manipulation in patients on long-term miotic therapy and in uveitic patients [4]. Following routine cataract surgery, Miyake et al [6] reported a 4.4% overall incidence of pupillary fibrin membrane formation in Japanese patients, typically around post-operative day five.

The pathophysiology of post-operative fibrin clots is thought to be secondary to a transient lowering of IOP and disruption in the blood-aqueous barrier during CE, resulting in leakage of fibrinogen-rich fluid from arterial plasma into the AC, eliciting a fibrinoid reaction [7–9]. If untreated, this fibrin can consolidate and result in a dense pupillary membrane. The low IOP seen in this patient was likely secondary to ciliary body shutdown in the setting of anterior uveitis.

To the best of our knowledge, this is the first report in the ophthalmic literature of a subacute post-operative anterior uveitic mass in such a well-circumscribed configuration that completely resolved after a short course of topical steroids. We surmise that this mass represents a fibrinous exudate in the setting of rebound iritis after cataract surgery involving a pupil expansion device. Ophthalmologists should be aware of this unique presentation after intraocular surgery.

## Data Availability

Not applicable.

## Abbreviations

CE: Cataract extraction
PCIOL: Posterior chamber intraocular lens
OD: Right eye
AC: Anterior chamber
VA: Visual acuity
IOP: Intraocular pressure
rAPD: relative afferent pupillary defect
DF: Descemet’s folds
